# NUS so fast: the social and ecological implications of a rapidly developing indigenous food economy in the Cape Town area

**DOI:** 10.1007/s11625-024-01528-z

**Published:** 2024-07-29

**Authors:** Jenny Willis, Bruno Losch, Laura M. Pereira

**Affiliations:** 1 Centre of Excellence in Food Security, University of the Western Cape, Cape Town, South Africa; 2 French Agricultural Research and International Cooperation Organization (CIRAD), Montpellier, France; 3 Global Change Institute, University of the Witwatersrand, Private Bag 3, Johannesburg, 2050 South Africa; 4 Stockholm Resilience Centre, Stockholm University, Stockholm, Sweden; 5 Centre for Food Policy, City University of London, London, UK

**Keywords:** Cape Floristic Region, Chefs, Food systems, Gastronomy, Neglected and underutilised species, Traditional ecological knowledge

## Abstract

The modern global food system is highly unsustainable, shaped by industrialisation and corporate consolidation, with negative repercussions on the environment and biodiversity as well as human health. This paper looks at the burgeoning economy in neglected and underutilised species (NUS) in the Western Cape, South Africa, as a potential innovation that could make the local food system more socially and ecologically resilient. Although, at present, NUS are only marginally included in the local food system and policy discussions, NUS increasingly appear in the high-end food industry, driven by international gastronomic trends. These species hold potential as climate resilient, nutritionally dense, and socially and culturally significant foods in the region, but they also carry ecological and social risks. We critically examine the fledgling NUS economy in the Cape Town area to unpack the motivations and challenges associated with the potentially transformative inclusion of these foods into the local food system. We demonstrate that the main risks associated with NUS are negative ecological repercussions, privatisation of the NUS economy, and the reproduction and further entrenchment of unequal power and race dynamics in the region. To mitigate these risks and actualise the related benefits associated with NUS, engagement with the ecological, social, and political context of NUS needs to be significantly deepened. This is particularly true for those working in the high-end food industry, who appear to be driving the NUS economy, and will require education around sustainability and Traditional Ecological Knowledge (TEK), as well as a foregrounding awareness of power dynamics.

## Introduction

The global food system is ecologically unsound and vulnerable to the impacts of the climate crisis, population demands, and economic shocks (Pereira 2017; Gordon et al. 2017). The number of people experiencing chronic hunger is rising, and the COVID-19 pandemic further intensified the vulnerability of global food systems, pushing hundreds of millions more into hunger (FAO 2020). Increasingly, the flow of food in this system is controlled by consolidated agrifood corporations, with supermarket shares growing in every world region to the exclusion of small producers, processors, traders, and retailers (Reardon et al. 2021). As a result of the industrialization and corporate concentration in the food system, dietary diversity has dwindled.

Regardless of country income, more diverse diets are now associated with high socioeconomic status (Mayen et al. 2014). Though diversity is a key component of adequate dietary intake and health, three-quarters of global food needs are now met by 12 plant and five animal species, whilst over half of global caloric needs are met by the three staple carbohydrates of rice, maize, and wheat (Gomez et al. 2013; Jaenicke and Höschle-Zeledon 2006). Meanwhile, many food species are neglected and underutilised. These neglected and underutilised species (NUS) are increasingly recognised for their potential as ecologically appropriate food crops that may help to improve climate resiliency and food security, diversify diets, and preserve food culture (Mbhenyane 2017; Akinola et al. 2020; Bioversity International 2017; Bacchetta et al. 2016; Coetzee 2018).

In South Africa’s Western Cape province, environmental and socioeconomic challenges continue to pressurise a food system already in crisis. The agricultural system, defined by scarce arable land and water, is highly vulnerable to the impacts of climate change (Pereira and Drimie 2016). Income and wealth inequality, split along racial lines, remain among the world's highest, and poverty is rising (SAHRC 2018). Though there is enough food to feed everyone adequately, low-income households struggle with malnutrition in the form of undernourishment, micronutrient deficiency, and obesity (Ledger 2016; May 2017). Processed, high-calorie and low-nutrition diets absent in diversity—‘Westernised diets’—are now common among low-income households (Battersby and Crush 2014). This ongoing nutrition transition, wherein traditional diets are replaced by the so-called Western diet (Popkin et al. 2012), is environmentally unsustainable, detrimental to health, and disconnected from ecological and cultural contexts. Furthermore, the impact of the breakdown of this system disproportionately affects the poor, Black people, and women (Kesselman 2023).

Though a more sustainable, ecologically and culturally appropriate food system is pressingly necessary in South Africa, indigenous NUS are only marginally included in agrifood systems and are largely absent from the food policy debate. In the Western Cape their use, however, is growing in the highly visible realm of fine dining, a key sector in the regional economy. The aim of this research was to investigate the potential of NUS to contribute to both the ecological and social resilience of food systems broadly by focussing on a unique case study exploring the growing economy of indigenous NUS in the Cape Town area, capital of the Western Cape and South Africa’s second most populous city.

## Literature review

As challenges to the food system mount, innovation around NUS will become increasingly important (Pereira et al. 2019). Literature on the role of chefs claims that by popularising NUS through restaurants and related food activities, chefs could be instrumental in mainstreaming biodiversity and dietary diversity and emerge as potential allies in these innovations (Bioversity International 2017; Pereira et al. 2019). An influential culinary movement seems tailored for this role: New Nordic cuisine, “wash[ing] through the world’s top kitchens” for more than a decade, often features NUS in the form of hyper-local and hyper-seasonal wild foods, whilst “engaging with agriculture and history and making age-old food delightful to modern palates” in a way that emphasises regional conditions and traditions (Moskin 2011, p. D1; Norden 2008).

Connected to the New Nordic culinary movement, foraging wild foods “is experiencing a gastronomic revival and is regaining significance in contemporary food culture, nutrition paradigms, local economies and society at large” (Munke et al. 2015, p. 206). The literature describes the potential for harnessing interest in NUS to “broaden culinary horizons,” whilst at the same time promoting the diversification of food and agriculture to build more ecologically and socially resilient, as well as nutritious, food systems (Munke et al. 2015, p. 206).

This culinary movement is now playing out in the Western Cape, where plants gathered by the region’s first inhabitants constitute the basket of locally adapted NUS beginning to appear in the province’s high-end food industry (Table 1). The NUS relevant to this study, based on their appearance in the Cape Town area’s high-end food industry, are represented in Table 1. It is important to note that in some cases, the plant part used in the food industry does not provide enough information to differentiate between morphologically similar species. Table 1 provides an overview of the relevant genera, noting the number of native species per genus as well as conservation statuses from the South African National Biodiversity Institute’s Red List of South African Plants database (SANBI).

**Table 1:** NUS appearing in the Cape Town area’s high-end food industry

	Common name	Scientific name	Description	SANBI conservation status per genus	
				Vulnerable to extinction	Least concern
Vegetable	Ice plant, *soutslaai*	*Mesembryanthemum* spp.	Terrestrial succulent, some with edible leaves, flowers and fruit	18	85
	Dune spinach	*Tetragonia decumbens*	Terrestrial coastal succulent with edible leaves	3	24
	*Veldkool, sandkool*	*Trachyandra* spp*.*	Terrestrial succulent, some with edible flower racemes similar to asparagus	13	47
	*Waterblommetjie*	*Aponogeton* spp.	Floating aquatic plant, some with edible leaves and flowers	3	6
	Sorel, *suring*	*Oxalis* spp.	Terrestrial herb, some with edible stems, leaves and flowers	70	133
	Samphire	*Salicornia* spp.	Terrestrial saltmarsh plant with edible stems		4
	Kelp	*Laminaria pallida, Ecklonia maxima*	Marine edible brown algae	No status available. *E. maxima* beds common from South Africa's West Coast to Nambia, *L. pallida* more widely distributed	
	Laver, *klipkombers*	*Porphyra capensis, Pyropia capensis*	Marine edible red algae	No status available. A number of cryptic species are found in South Africa, molecularly differentiated from *Porphyra* and *Pyropia* found elsewhere globally (Reddy et al. 2018)	
Aromatic	*Buchu*	*Agathosma* spp.	Terrestrial shrublet, some with edible leaves and flowers	90	55
	Cape may	*Coleonema* spp.	Terrestrial shrublet, some with edible leaves and flowers	2	6
	Wild sage	*Salvia* spp.	Terrestrial shrub, some with edible leaves and flowers	2	21
	Wild rosemary, *kapokbos*	*Eriocephalus* spp.	Terrestrial shrub, some with edible leaves and flowers	4	28
	Wild garlic	*Tulbaghia* spp.	Terrestrial herb, some with edible leaves and flowers	5	16
	Dune celery	*Dasispermum suffriticosum*	Terrestrial herb with edible leaves and flowers	1	5
	Searsia	*Searsia* spp.	Terrestrial shrubby tree, some with edible seeds similar to sumac	11	65
	Pelargonium, geranium	*Pelargonium* spp.	Terrestrial herb, some with edible leaves and flowers	50	179
	Wild mint	*Mentha* spp.	Terrestrial herb, some with edible leaves and flowers		4
Fruit	Sour fig, *suurvy*	*Carpobrotus* spp.	Terrestrial succulent, some with edible fruit	1	7
	*Slangbessie*	*Lycium* spp*.*	Terrestrial shrub, some with edible berries		22

Globally, wild food and NUS are increasingly linked to local culture and identity (Munke et al. 2015; Bioversity International 2017; Owns and Shackleton 2018; Bacchetta et al. 2016). They can serve as cultural keystones for local communities who traditionally used wild foods, forming “part of a living link with the land” (Bharucha and Pretty 2010, p. 2922).

Applying this lens to the Western Cape requires consideration of ecological and social context. Situated at the southernmost tip of the African continent, the Western Cape contains much of the Cape Floristic Region (CFR). The CFR is the smallest and most diverse of the world’s six Floral Kingdoms, uniquely rich in species adapted to the province’s largely nutrient poor and acidic soils (Manning 2013; Valente et al. 2014), with an extensive history of wild plant use for food and medicine. More than 1000 indigenous edible plant species have been documented over the last 300 years, an accumulation of knowledge largely gleaned from observations of the Khoikhoin and San first peoples who relied on these species (DeVynck et al. 2016; Philander 2010).

In the social context of the CFR, much of the traditional ecological knowledge (TEK^1^) surrounding the use of native NUS has been eroded (Bvenura and Afolayan 2015). Colonial expansion beginning in the 1700s led to the “collapse” of the Khoikhoin and San peoples, and much of their hunter–gatherer tradition was consequently lost (Philander 2010; DeVynck et al. 2016). Marginalisation and the institutionalisation of segregation through apartheid subsequently engineered further rejection of heritage and identity among the remaining descendants of the Khoikhoin and San, enforcing simplistic racial categories—Black, Coloured,^2^ and Indian—to keep non-white South Africans from forming alliances that would threaten apartheid rule (Philander 2010; Western 1997).

Since wild-harvested products are typically accessed through TEK passed down through generations (Munke et al. 2015), negation of Khoikhoin and San identity contributed to the ongoing erosion of knowledge around the use of native NUS in South Africa. The Group Areas Acts of the 1950s and 1960s, which established forced population removals according to apartheid racial mapping, further separated people from their connection to land, uprooting and displacing descendants of the Khoikhoin and San throughout the Western Cape. If TEK has the ability to “connect the present to the past and reestablish resilience” (Gunderson et al. 1997 in Berkes et al. 2000, p. 1260), then reclaiming and even regenerating TEK around NUS could have a role in addressing the multiple histories of displacement from Indigenous identity in the Cape Town area. Applying a framework of decolonization to this question of NUS in the Western Cape could therefore have the potential to create a space to challenge the hegemony of the current (neo)colonial/capitalist food system and replace it with the values of reciprocity, collectivity, and inter-connectedness that underpinned pre-colonial food systems with implications for other colonised food systems (Kesselman 2023).

Besides potential cultural significance, NUS could contribute to dietary diversity and food security in the Cape Town area, where the food system is characterised by unacceptably high rates of food insecurity (Crush et al. 2018). NUS are often nutritionally dense and micronutrient-rich foods, higher in micronutrients and metabolites than equivalent cultivated foods (Akinola et al. 2020; iPES-Food 2016; Bacchetta et al. 2016, p. 181). Because they are naturally adapted to the CFR, certain NUS could be grown with minimal inputs and irrigation.

Conventional agriculture, as well as urbanisation, encroachment by alien species, and climate change, threaten the CFR’s unique ecoregions (Roura-Pascual et al. 2009; Gaertner et al. 2012) Because much of the soil in the province is considered poor and freshwater resources are scarce, conventional agriculture requires various interventions, including liming, the application of gypsum, irrigation, artificial drainage and tillage practices, and other strategies (Tembo-phiri 2019, p. 21). Conversely, the CFR’s soils support the species adapted to them without the need to apply excessive energy or other resources (Tembo-phiri 2019). As ecologically appropriate species, indigenous NUS may be suited for more climate-resilient farming in the Western Cape, where severe drought from 2014 to 2019 applied even greater pressure to the region’s limited freshwater resources.

Given the region’s unique ecology and history, driving the use of indigenous NUS as food poses certain risks. Wild harvesting in the CFR, where many vegetation types and microbiomes are threatened, on the verge of extinction, or already extinct, may lead to further ecosystem damage. The ecological repercussions of monocrop cultivation and seed cross-contamination in a region with so many microhabitats and subspecies must also be mitigated. Furthermore, the development of the NUS sector within the present context of inequality in South Africa could entrench historically established power dynamics (Müller et al. 2016), to the detriment of the people previously reliant on NUS or those who possess TEK (Pereira 2017). This is particularly important to consider in the South African context, where inequality rates are among the world’s highest and colonialism, apartheid, and the development of an industrialised food system have contributed to the erasure of TEK belonging to people with Indigenous heritage.

## Methodology

This study used explorative qualitative study design and content analysis, allowing for a fine-grained, context-specific exploration of the emerging NUS economy in the Cape Town area, which is situated within the unique CFR biome. Whilst the findings are specific to this context, the broader aspects of a decolonial praxis in seeking food system transformation through the inclusion of NUS have implications outside of the case study area. Fieldwork was conducted through participatory observation and in-depth interviews. All interviewees gave their written informed consent, including for the recording of interviews, and the research protocol was cleared by the Research Ethics Committee of the University of the Western Cape.

Participatory observation took place at two ‘wild food’ workshops, hosted by a prominent stakeholder in the NUS economy and intended to engage chefs, growers, and those with roots in the CFR^3^ in inclusive dialogue around the use of NUS in the local food industry. These workshops served as a first investigation to identify the stakeholders engaged with NUS in the Cape Town area (see Pereira et al. 2022 for more information). The embeddedness of the lead author in the local food industry over a period of 4 years was a significant asset; this experience included work with a chef leading the gastronomic revival of NUS in the area, and then with two Cape Town social enterprises supporting market access for small-scale farmers and fishers through relationship building with chefs and restaurants. These years of de facto preliminary investigation facilitated observations and first-hand knowledge about what was occurring around NUS and who was participating in the NUS economy, whilst establishing relationships throughout the local food system.

Between August 2018 and February 2019, 17 open-ended, semi-structured interviews were conducted with stakeholders in the NUS economy, selected through purposive sampling. Interviewee profiles are listed in Table 2. The interviewee spread was designed, so that all roles in the economy would be represented: chefs, bartenders, educators, growers, producers, government agency representatives, and suppliers. A number of interviewees straddled multiple roles, whilst some roles were limited to only a few interviewees. Given the racial history of South Africa, we have included the race and nationality of the interviewees, which emphasise the racial divide between growers and users of NUS. The lead author’s embeddedness in the local food industry will have influenced what certain interviewees decided to discuss; an attempt to address this was made through the use of neutral, non-judgmental language and by allowing conversations to proceed organically. The same general questions were posed to all interviewees, regarding the motivations and challenges they were encountering or noticing in the economy as it was emerging, with follow-up topics and questions covered as they naturally occurred. The interviewee list is not meant to be a comprehensive sample of all stakeholder perspectives on the NUS economy, but, together with the other data, to present diverse perspectives from leading figures in the industry (kept anonymous for ethical reasons).

**Table 2:** Interviewees’ profiles

	Role	Secondary activity	Status	Engagement with NUS	Gender	Age	Racial self-identification
1	Grower	Supplier	Self-employed	< 5 years	M	30s	South African of colour
2	Grower	Aspiring supplier	Community leader, self-employed	Roots in the CFR (and elsewhere)	M	40s	South African of colour
3	Aspiring grower	Aspiring supplier	Community leader, self-employed	Roots in the CFR (and elsewhere)	F	50s	South African of colour
4	Grower	Supplier	Self-employed	< 2 years	F	30s	Non-South African of colour
5	Chef		Restaurant owner	< 4 years	M	40s	White South African
6	Chef	Aspiring grower	Restaurant employee	< 1 years	F	30s	White South African
7	Chef		Restaurant employee (colleague of interviewee 8)	< 1 year	F	20s	White South African
8	Bartender		Restaurant employee (colleague of interviewee 7)	< 1 year	F	20s	White non-South African
9	Chef		Restaurant owner	< 2 years	M	30s	White South African
10	Chef		Restaurant owner	> 10 years	M	30s	White South African
11	Supplier	Grower	Business employee	< 2 years	M	30s	White South African
12	Supplier		Non-profit staff member	< 1 year	F	40s	White South African
13	Supplier	Educator	Self-employed	> 7 years	M	30s	White South African
14	Educator	Supplier	Self-employed	Roots in the CFR (and elsewhere)	F	30s	South African of colour
15	Educator	Supplier	Self-employed	> 10 years	F	60s	White South African
16	Educator	Artist	Self-employed	Roots in the CFR (and elsewhere)	F	30s	South African of colour
17	Government agency representative		Government employee	Roots in the CFR (and elsewhere)	M	30s	South African of colour

Qualitative content analysis was used to categorize and cluster the collected data so that patterns and themes could be identified. The seventeen interviews were transcribed by hand, iteratively, so as to further immerse the lead author in the data. Noting keywords and recurring concepts in the process of coding, passages were often reworked as connections emerged and solidified between interviews. These codes were then grouped into categories which were gradually refined and condensed, ultimately synthesizing the findings into manageable components. A longer discussion of the methods and analysis, together with all of the results can be found in Willis (2020).

## Results: investigating NUS in the Cape Town area high-end food industry

### A dearth of knowledge

Most interviewees identified a lack of knowledge related to NUS as a constraint in the emerging NUS economy. Yet, there were substantive differences in how interviewees framed this issue, falling into two main groups. The first group, representing a majority of interviewees in the food industry, described the problem in terms of their access to information.

Interviewees in this group described their need for more information on culinary applications and seasonality, commenting on the irony of local culinary schools teaching foreign cuisines, but nothing about the use of indigenous NUS. These interviewees wanted this information, so that they could use NUS “correctly”, both in terms of producing appetizing food and avoiding food safety issues. Only one interviewee in this group commented on the connection between knowledge around NUS and TEK, noting “*We still don’t have as much information about the traditional uses… the people are keeping most of this info secret*.”

In the second group (just more than half of the interviewees), the lack of knowledge around NUS was attributed variously to the erasure of TEK by colonialism, apartheid and globalisation, rather than as an issue with accessing information. Local people’s TEK had been “*stunted, because people got displaced from land,*” said a white chef in this group. A government agency representative with roots in the CFR described how “*around Cape Town there’s a disturbance halo*” caused by forced removals: there is little TEK within this halo, because it is too painful for people to think about the homes and land they lost. Whilst more TEK remains among older people living in rural areas outside this halo, disinterest from the young was causing further loss. An educator with roots in the CFR attributed loss of TEK to environmental damage, in that species once readily accessed as food may now be extinct or critically endangered, making them difficult or impossible to access and rendering TEK around their use obsolete.

Several interviewees in the second group were grappling with ownership and possession of TEK. Who has the right to evolve or adapt TEK? Who has the right to profit from it? Who has the right to share it, and what are the distinctions between sharing, influencing, inspiring, gleaning, taking, and stealing (relevant especially in the context of social media)? These questions were overlaid by complex tensions around race and culture in South Africa. A white supplier described positionality in the emerging NUS economy as “*a minefield,*” particularly where people with roots in the CFR who do not possess knowledge related to NUS—perhaps as consequence of the systematic erasure of TEK—would like to engage in the growing niche economy. How is this to be navigated, especially where peoples’ “*ancestors might have harvested [NUS] but they haven’t, and I don't think they have a lot of direct knowledge themselves*”? Two white educator-suppliers spoke about “*waking up*” to the sacredness of the knowledge that had been shared by people with “*a much more firmly rooted history in this nation,*” or being “*educat[ed] in how to speak*” from people with more “*deeply connected positions… from an inside place*” in the CFR.

### Limited access

The emerging NUS economy is limited by physical access issues. Main comments had to do with consistency, quality, sufficient quantities, and the generally limited availability of NUS. “*Where is my access to these plants? I still don't have them. I need it. I’m ready,*” said one white chef.

Several suppliers, growers, and aspiring growers found it challenging to obtain seed or plant material for growing NUS native to the CFR. One white supplier joked that he was so stymied by where to find cultivation material that he might “*go and steal some cuttings from [a research centre] over the weekend.*” This supplier had explored importing seed but was discouraged by the onerous legal process, whilst a grower had sourced seed from the United States informally.

Many interviewees shared concerns about commercial development and exploitation of NUS. For most, the primary concern was that entities with capital and resources would develop the market before smaller businesses or communities with roots in the CFR could engage in the economy: capital, land tenure, market access, and financial management skills were noted as related challenges. A government agency representative with roots in the CFR was concerned that commercial interests would pursue an extractive monoculture model, with potentially irreversible repercussions on the CFR through the clearing of intact ecosystems, whilst a white supplier was concerned about the ecological repercussions of cultivating NUS from foreign seed (which may not correspond genetically with locally specific species), in light of practices he had observed among growers of NUS from whom he sourced.

One educator with roots in the CFR described her work with corporate clients as “*purely commercial and extractive*”, whilst a supplier of NUS to the food industry described his decision to close his wild-harvested NUS business in part because of his concern that others could approach the economy as “*a get rich scheme*”. That commercial development of NUS is already underway eventually emerged through the interviews, though only one supplier understood the scale of cultivation currently supplying the bulk of NUS to the food industry: most NUS available in the Cape Town area comes from a commercial grower in Gauteng, more than 1000 km from the CFR. This company was described as using artificial seawater, electric lighting systems, and tunnels to cultivate imported seed.

### Notions of sustainability

Though the sustainability of NUS was a consistent theme among interviewees, again two varying perspectives emerged. The primary concern for half the interviewees was that valorization of NUS could lead to, or was already leading to, unsustainable wild harvesting. A white supplier described illegal harvesting in conservation areas, where people were “*pillaging dunes and destroying the environment*.” The environmental impact of hobbyist foragers was a concern, particularly for educators: one educator with roots in the CFR described how attendees of her workshops often arrived saying “*‘I want to go up Table Mountain on my walk and be able to collect things and eat them*,” apparently unaware of sustainability issues or conservation regulations.

Wild harvesting regulations were generally considered opaque. One supplier suspected that his business was “*dangerously bordering on illegal harvesting*.” Other interviewees expressed their frustration with the regulation process, particularly regarding the commercial orientation of governance, which can prohibit the involvement of small or community-based entities. Government tends to engage with existing commercial entities because of their established and “*recognisable structures*,” said a government agency representative, in contrast to communities that can sometimes be internally fragmented or “*a little bit dysfunctional in terms of admin*.”

Most of the interviewees concerned about wild harvesting in the CFR viewed cultivation as a sustainable alternative. Some also believed that NUS could be cultivated as a more sustainable alternative to conventional agriculture in the region: grown as more ecologically appropriate, climate-resilient crops, requiring less water and fewer inputs. Several interviewees noted that it “*makes total sense*” for local farmers to be growing NUS, because they are “*climatically easier,*” less water intensive and therefore adapted to aridity and drought. One government agency representative with roots in the CFR was interested in the agroecological cultivation of NUS as a means to restore degraded landscapes.

In contrast to these views, a majority of interviewees in the food industry expressed the view that NUS were “*very sustainable*” to use “*because they are everywhere*” and “*super local*”. Using local NUS in place of imported ingredients with high carbon footprints was also a sustainability motivation for one white chef.

### Gastronomic dilemmas

The use of NUS was widely recognised as a popular gastronomic trend rapidly gaining local traction among tourists and the high-income segment of the Western Cape. Interviewees felt that the use of NUS had “*taken off*” and become “*a cool thing*” to the point of ubiquity among the region’s top restaurants. A few interviewees attributed this to a trickle-down of gastronomic culture from Europe, whilst others considered the restaurant *Wolfgat* in the town of Paternoster (the ‘Best Restaurant in Africa 2021’, according to the World’s 50 Best Restaurants (2021) to have influenced the rising popularity of NUS in the region’s food industry.

The trend concerned some interviewees. One white supplier expressed concern about building a supply chain around potentially fickle demand for NUS. Other interviewees felt that the trend was leading to superficial use of NUS, stripping the food of cultural and ecological meaning. Superficial engagement disregards the significance of NUS as foraged foods in pre-colonial times: “*because it's blowing up, because of the chef scene, you bypass all of [the meaning] so that it doesn’t have the nostalgia and memory and the story…*” said one educator with roots in the CFR. “*You’ve lost the spirit, the cultural and spiritual ecology.*” A grower compared the use of NUS in high-end restaurants to the Natives Land Act of 1913, which restricted Black South Africans to reserves and allocated 80% of arable land in South Africa to the minority white population: there is “*no difference in the activities of 1913 and this. It is the same result for food culture: it’s [a] slap[ped] in the face…*”.

A majority of stakeholders in the food industry said that they were interested in using NUS, because they are unique, interesting, and exciting ingredients. NUS were described as more fun to use than conventional ingredients, as “*completely unique and foreign*”. One white chef explained that ingredients like NUS that “*other people can’t easily get hold of or don't know how to handle*” are what creates excitement and gives a restaurant an edge in a challenging industry.

Exclusivity was recognised as a challenge in the NUS economy as it stands: use at high-end restaurants was seen by one grower as “*the new way of colonizing those foods,*” whilst a white supplier described growing discomfort with “*taking this Indigenous knowledge and giving it to these white restaurants,*” ultimately “*supplying high-end chefs when most South Africans can’t afford to eat*.” Some chefs found NUS too expensive—“*the prices are insane,*” said one—whilst others explained that expensive NUS ingredients were a *“draw factor*” worth building into the cost of high-end dishes. There was hope that NUS would become more normalized, appearing in less exclusive spaces where not only wealthy diners would be exposed to them. For this to happen and for NUS to become less exclusive, said one educator with roots in the CFR, there is a need for *“all the invisible blocks*” attached to NUS to be dismantled, in addition to making them more affordable and widely available. The example of such an “*invisible block*” was the positioning of NUS as substitutions for Japanese ingredients, which limits the relevance of NUS to people familiar with Japanese cuisine.

Negative stigmas attached to NUS as well as food aspirations linked to globalisation emerged as key challenges in the NUS economy. Contrary to discussions about exclusivity, one grower with roots in the CFR described how NUS had become emblematic of poverty in her more rural community, only eaten as a “*last resort because there’s no money to buy chicken or meat*.” One white chef also noted links between poverty and NUS, describing how *“it’s looked down upon and mostly seen as a poor man’s thing if you forage. There’s almost pride attached to the fact that you can afford to buy from the supermarket*.” This connection between poverty and foraging for NUS was echoed by an educator with roots in the CFR: “*it’s seen as something one would do if you’re very, very poor*.”

## Discussion

Though NUS have potential to contribute to the ecological and social resilience of food systems, their use in the Cape Town area’s food industry is complicated by issues of sustainability, aspiration, exclusivity, and power. Applying a decolonial framework to this challenge of NUS in food systems opens up important conversations around colonial legacies, race, displacement, marginalisation, right, dignity, and the recognition of knowledge systems that are important factors to consider in the fledgling NUS economy.

### Sustainability and the contradictions of the NUS economy

There are several sustainability concerns in the NUS economy as it currently stands in the Cape Town area. Because of the region’s concentration of biodiversity and threatened ecological status, even careful agroecological farming practices in the CFR may lead to ecosystem damage and will have to be rigorously monitored to prevent negative repercussions. However, a scarcity of available seed and plant material for cultivation is already pushing stakeholders to purchase seed from companies in the United States and Europe.

This imported seed may have unintended consequences in the CFR, with its unique microhabitats and vulnerable endemic species. Samphire is such an example: appearing regularly in the high-end food industry, the common name samphire refers to a genus of halophytic succulents (*Salicornia*) occurring in Europe, North America, southern Africa, and south Asia, with extremely localized subspecies native to the CFR (Slenzka et al. 2013)*.* Because seed for local subspecies is not available, growers are importing seed for foreign *Salicornia* species. Genetic infiltration of wild populations is then possible, with irreversible implications on local species.

This study also found that despite evidence that sustainability was a strong motivation for engagement with NUS, the concept of sustainability was only partially understood by key stakeholders. For some, NUS were considered sustainable because of their local adaptation to the region’s soil and climate: NUS could potentially diversify the agricultural production system, which currently focuses on the conventional crops that require intensive inputs and irrigation, and be used to develop a more resilient agriculture for the Western Cape. This echoes the potential of NUS to build resilience through farming system diversity and agrobiodiversity (Pereira 2017; iPES-Food 2016; Bacchetta et al. 2016).

Others, predominantly in the food industry, considered NUS sustainable because of their perceived ability to grow “*everywhere*.” This perception justified wild harvesting, without acknowledging the associated risks, such as ecosystem damage or the challenge of correctly identifying morphologically similar species within genera including a number of threatened species, as shown in Table 1, or developing the notion into an argument for cultivation of NUS as ecologically appropriate crops. Some in the food industry also described a preference for the flavour of wild-harvested NUS over cultivated. The potential environmental repercussions of the NUS economy need to be better understood throughout the food system, but particularly among those in the food industry most visibly driving the market for NUS. This is especially relevant in the CFR, where wild harvesting as well as cultivation may cause damage in already precarious ecosystems.

### Health and food security vs reality and practice

Advocates posit that NUS crops could be grown for subsistence or market, with the potential to contribute directly to food security as home-grown, nutrient-rich foods or indirectly to food security through income generated by market gardening. Because they require fewer inputs than conventional crops and minimal irrigation—and therefore lower investments and production costs—certain NUS could be grown relatively easily in the Cape Town area and, it follows, across Cape Town’s food insecure townships both for subsistence and for market.

Ease of cultivation was anecdotally corroborated by two growers of NUS in this study, but there is a critical dualism in the NUS economy that complicates this picture. On the one hand, people are reluctant to eat NUS due to negative stigmas, aspiration, and the nutrition transition. On the other hand, in the food industry frequented by wealthier people or foreign visitors, NUS are highly valued, sought-after ingredients. This dualism, as well as issues of limited access, combines additively to prevent NUS from contributing meaningfully towards local food security.

Negative stigmas around NUS were seen by a number of stakeholders as limiting engagement with NUS in poorer Cape Town communities, echoing findings on negative perceptions attached to NUS from two recent studies in the area (Du Bruin 2018; Kelly 2016). These stigmas were connected to aspirations signifying connectivity to the global food system, reflecting the aspirational nature of purchasing global food system staples like wheat and rice (Pereira 2017; Puoane et al. 2006) and supporting Kelly’s finding that “aspirations towards modern, urban lifestyles reflect a disregard and loss of knowledge about wild edible plants” in the Western Cape (Kelly 2016, p. 4).

These aspirations towards globalised foods correlate with South Africa’s ongoing nutrition transition, in which traditional foods and consumption patterns are replaced by a ‘Western’ or ‘modern’ diet high in refined carbohydrates, sugar, fat, and animal-source foods (Popkin et al. 2012; Lang and David 2012). This is not a choice: over millennia humans have evolved biochemical and physiological processes that value fat and sugar, meaning that humans have biologically wired preferences that make them susceptible to the high fat and high-sugar foods of the nutrition transition (Popkin 2017). The nutrition transition and stigmas attached to NUS underlie the perception, evident in this study, of modern palates as transformed to such a degree that beyond disinterest in NUS, many people no longer find NUS acceptable as food.

Conversely, usage of NUS in the high-end food industry contributes to their exclusivity, further limiting their potential contribution to health and food security. For one, cost is prohibitive to the food insecure. Sold by only a handful of suppliers, NUS are currently marketed as specialty goods: considered by some chefs too expensive even for the margins of their fine dining establishments, or by others as ingredients whose high cost must be built into the overall high prices of their menu. Second, NUS are valued in the food industry for their novelty, or their status as special, niche, or difficult-to-come-by ingredients. This valuation requires NUS to remain novel or niche, rather than mainstream or accessible. This works at cross purposes with the potential of NUS to improve dietary diversity for the food insecure (Mabhaudhi et al. 2018; Mbhenyane 2017), which would require expanding and encouraging their everyday use rather than reinforcing their rarity. This is not a problem unique to the CFR, but to mainstreaming access to NUS more generally.

Scarcity, or limited access, emerged as an additional challenge preventing the meaningful contribution of NUS to health and food security. First, issues of access must be considered in the CFR’s context of environmental disruption, where wilderness is largely either degraded or conserved—both of which make it difficult for people to access NUS in the traditional ways. Next, demand for NUS is currently greater than available supply. Though there is reticence to eat NUS in poorer communities, NUS are increasingly sought after in the region’s high-end food industry, but the existing supply chain is characterized by issues with consistency, quality, and quantity.

Further limiting the NUS economy is the lack of knowledge on cultivation practice and material for cultivation, curtailing prospective growers interested in contributing to the supply chain. The legal landscape around any potential sustainable wild harvesting is characterized as unclear and onerous, limiting the development of a supply chain based on wild NUS. This scarcity is frustrating many in the food industry, who want better and more reliable access to NUS. However, in one way, it serves the industry by maintaining NUS’ status as novel, hard-to-come-by ingredients.

### Identity, culture, and power

In this dualistic context of stigmatization and high-end valuation, NUS were also recognised as a means to reconnect with heritage and culture eroded over time by colonialism, apartheid and the development of an industrialised food system. However, the historical rejection of heritage and identity among descendants of the Khoikhoin and San complicates the analysis of such reconnection in the Western Cape: none of the interviewees, for example, identified explicitly with Khoikhoi or San ancestry. Instead, the term “roots in the CFR” was formulated with input from interviewees who identify as Coloured as a designation more inclusive of the ambiguity around Coloured and Khoikhoi and San identity formation in the Western Cape. Among those with roots in the CFR as such, or with Indigenous and non-European roots elsewhere in South Africa, engagement with NUS was considered a tool that can help to make sense of the complexity around identity, indigeneity and belonging, with the potential to help facilitate healing. This is an encouraging reflection of the literature positioning NUS and the related TEK as an important linkage between people and land, capable of “defining biocultural identity” (Bioversity International 2017, p. 34). However, these stakeholders were also concerned about the appropriation of NUS.

Considering the systemic erasure of TEK in the Cape Town area, this valuation of NUS draws on TEK’s ability to connect to the past and establish resilience (Gunderson et al. 1997 in Berkes et al. 2000, p. 1260), and its potential as “a tool of mental archeology” in development practice, particularly valuable in contexts of entrenched and unequal power dynamics (Haider and Oudenhoven 2018, p. 6). However, for the potential of NUS as socially and culturally significant foods to be actualised in the Cape Town area, more stakeholders would need to engage with the history of NUS usage in the CFR and the role of TEK. Only half of those interviewed in this study connected knowledge around NUS to TEK, or articulated the factors behind the decline in TEK related to indigenous NUS. Interviewees in the food industry, particularly, did not make this connection, indicating the risk of appropriation by some stakeholders, and highlighting the potential need for a decolonial approach.

It must be acknowledged that whilst decolonial thinking in the sustainability sciences is relatively recent, except for scholars like Escobar who have written extensively on this for decades (Escobar 2000, 2015, 2018, 2020), much of the broader engagement with decoloniality has been led from the Americas (Maldonado-Torres and Cavooris 2017; Mignolo 2021; Mignolo and Escobar 2013; Quijano 2007). This leaves a gap, that is slowly being addressed, on what decoloniality entails in an African context. Kesselman (2023) writes on the need for decolonisation of the South African food system to address historical injustices. The growing NUS economy in the Western Cape provides an important case through which to analyse how a decolonial praxis would foreground all the concerns raised by the stakeholders in the system and potentially open up ways to overcome some of the obstacles for equitable food system transformation that this research uncovers. A key aspect of this is the unequal distribution of power that is historically embedded.

In this study, power was weighted towards those in the food industry and supplier roles: these stakeholders were white, occupying positions of relative power as formal business owners or employees in management and leadership roles. Highly successful stakeholders in the food industry wield additional power through celebrity and the amplification of their voices through social media. Attention has been paid to the potential for good that those in the food industry can do with this power—“chefs today wield unprecedented global influence, which creates an opportunity for them to be effective advocates on issues that affect society far beyond the walls of their restaurants” (Cummings 2016: online)—but in reality, as demonstrated in this research, there is equal potential for that influence to reinforce existing power imbalances or encourage unsustainable practices like wild harvesting. This critical stance is important to account for in all case studies involving the role of chefs as potential changemakers to enable food system transformations, especially in the Global South or where indigenous knowledge holders are involved as the custodians of NUS (Pereira et al. 2019; Zocchi and Fontefrancesco 2020; Zocchi et al. 2021; Cherro Osorio et al. 2022).

In Cape Town, development of NUS is already falling into the conventional agricultural production modes thereby reproducing colonial power relations. The primary producer of NUS for the Cape Town area food industry is, as emerged in this study, a white-owned business located in Gauteng and exporting “to more than ten countries world-wide” (Pico Gro 2019). Whilst the Nagoya Protocol on Access and Benefit Sharing, ratified by South Africa in 2013, could be applied to the NUS economy, tensions around the custodianship of indigenous knowledge are likely to play out between groups linked to descendants of the Khoikhoin and San first peoples, as well as those involved in the NUS economy as it stands.

## Conclusion

The findings from this study are timely, not only for South Africa, but for other food systems with potentially transformative NUS (Zocchi and Fontefrancesco 2020; Zocchi et al. 2021; Cherro Osorio et al. 2022). Learnings about the historical legacy of power, erosion of knowledge systems and the need to transform food systems to be more locally adaptive, whilst meeting food needs equally is not a challenge unique to South Africa, but plays out globally. Whilst NUS hold potential as ecologically appropriate, nutritionally dense and culturally significant foods, the burgeoning NUS economy will require at the very least an acknowledgement of historical injustices, or even better a decolonial praxis that centres restorative justice and equitable participation before it is able to contribute materially to the ecological or social resilience of local food systems: who is to lead this movement, and on whose behalf, is unclear in the Western Cape.

Despite international activity around harnessing the influence of chefs to advocate for more sustainable food systems, this study has shown that the gastronomic industry in the Cape Town area is not presently equipped to advocate on issues related to NUS. Only one chef in this study expressed awareness of the potential for NUS to contribute to biodiversity, dietary diversity, or the development of an alternative agriculture for the Western Cape. Disconnected from these bigger picture considerations, it seems unlikely that chefs will lead lasting, positive change in the area and may in fact play a detrimental role. This is another important consideration for similar processes happening elsewhere, such as Mexico (Pereira et al. 2019), and should be taken as a serious knowledge gap that needs to be addressed.

Contrary to the initial hypothesis, chefs may in fact be positioned counter to the goal of responsibly mainstreaming NUS: instead of championing a gastronomic trend in the interest of increased biodiversity, dietary diversity, and sustainability, many were focussed on preserving the novelty or scarcity of NUS ingredients. In the shorter term, if the value of NUS is located primarily in their status as novel ingredients, then it follows that as NUS become more readily available, these ingredients will lose their appeal. In the longer term, positioning NUS in the high-end market may lead to their adoption as an elite or aspirational food, driving demand from the wealthy. The explicit engagement and sensitisation of chefs around these potential outcomes, as well as the related ecological and social risks, will be necessary to ensure that the gastronomic revival of NUS does not become another means to reproduce entrenched power dynamics.

This study has demonstrated that although NUS are only marginally included in the Western Cape’s agrifood system, the high-end food industry in the Cape Town area is driving the development of an emerging and fragile NUS economy. If the NUS economy continues to develop without a deepening of stakeholder conceptions of sustainability and the cultural significance of NUS native to the CFR within a decolonial praxis, it is likely to result in ecosystem damage both through wild harvesting and unsound agricultural practices. Whilst reclaiming and even regenerating TEK around NUS could help to address displacement from Indigenous identity in the Cape Town area, a revival of NUS that excludes the recognition of TEK risks reinforcing the marginalisation of Indigenous knowledge and identity. Unequal power dynamics, deeply entrenched in South Africa, appear to be shaping the NUS economy. These dynamics must be shifted, and stakeholder conceptions of NUS deepened on a number of levels, from sustainability considerations in the CFR to recognition of TEK. Privatisation of the NUS economy is already a concern: most of the NUS available to restaurants in the Cape Town area are grown by an agribusiness in South Africa’s Gauteng province. Similarly, the potential for NUS to contribute dietary diversity and nutrition for the food insecure can only be realised if efforts are made to surmount the stigmas, as well as the barriers of exclusivity, that are attached to NUS in different contexts.

The inclusion of NUS into the food security, food system, and sustainability debates globally opens up new spaces for discussion about development trajectories in other post-colonial spaces. A just food system transformation with NUS at its heart requires involvement of local, provincial, and national governments, as well as political will. More broadly, these findings allow us to dig more deeply into the framing of food as a tool for resilience building and chefs as important changemakers in challenging contexts where unequal power dynamics are deeply entrenched.

## Data Availability

All data used in this paper are available in the MSc thesis, Willis (2020).

## Footnotes

1 TEK is defined by Berkes et al. (2000, p. 1252) as “a cumulative body of knowledge, practice, and belief, evolving by adaptive processes and handed down through generations by cultural transmission, about the relationship of living beings (including humans) with one another and their environment”, and is particularly relevant to food systems, underpinning agrobiodiversity and potentially contributing positively to nutrition and health.

2 A racial designation in South Africa denoting a person of multiracial European (“white”) and African (“black”) or Asian ancestry, as officially defined by the South African government from 1950 to 1991. ‘Coloured’ therefore forms a multiracial ethnic group native to Southern Africa, having ancestry from more than one of the various populations inhabiting the region, including Khoisan, Bantu, Whites, Austronesian, East Asian, and/or South Asian. In the Western Cape of South Africa, a distinctive Cape Coloured and affiliated Cape Malay culture developed, whereas in other parts of the country, people classified as Coloured were usually the descendants of individuals from two distinct ethnicities.

3 The phrase “roots in the CFR” was chosen with input from multiple interviewees who identify as Coloured and is intended to be more inclusive of the complex nature of Coloured and Indigenous identity formation in the Western Cape.
